# Optimal Glomerular Filtration Rate Equations for Various Age Groups, Disease Conditions and Ethnicities in Asia: A Systematic Review

**DOI:** 10.3390/jcm12051822

**Published:** 2023-02-24

**Authors:** Aqsa Safdar, Waqas Akram, Mahtab Ahmad Khan, Sajjad Muhammad

**Affiliations:** 1 Faculty of Pharmaceutical Sciences, University of Central Punjab, Lahore 54000, Pakistan; 2 Department of Neurosurgery, University of Helsinki and Helsinki University Hospital, 00290 Helsinki, Finland; 3 Department of Neurosurgery, Medical Faculty and University Hospital Düsseldorf, Heinrich-Heine-Universität Düsseldorf, 40225 Düsseldorf, Germany

**Keywords:** Asian population, chronic kidney disease, CKD-EPI equation, creatinine, cystatin C-based equations, estimated glomerular filtration rate

## Abstract

**(1) Background**: The performance of estimated glomerular filtration rate (eGFR) equations in the Asian population has been widely questioned. The primary objective of this study was to gather evidence regarding optimal GFR equations in Asia for various age groups, disease conditions, and ethnicities. The secondary objective was to see whether the equations based on the combination of creatinine and cystatin C biomarkers if employed are satisfactory across different age groups and disease conditions in various ethnicities in Asia compared to those based on either of the single biomarkers. **(2) Methods**: Validation studies that had both creatinine and cystatin C-based equations either alone or in combination, validated in specific disease conditions, and those which compared the performance of these equations with exogenous markers were eligible only. The bias, precision, and 30% accuracy (P30) of each equation were recorded accordingly. **(3) Results**: Twenty-one studies consisting of 11,371 participants were included and 54 equations were extracted. The bias, precision, and P30 accuracies of the equations ranged from −14.54 to 9.96 mL/min/1.73 m^2^, 1.61 to 59.85 mL/min/1.73 m^2^, and 4.7% to 96.10%. The highest values of P30 accuracies were found for the JSN-CKDI equation (96.10%) in Chinese adult renal transplant recipients, for the BIS-2 equation (94.5%) in Chinese elderly CKD patients, and Filler equation (93.70%) also in Chinese adult renal transplant recipients. **(4) Conclusions**: Optimal equations were identified accordingly and it was proven that combination biomarker equations are more precise and accurate in most of the age groups and disease conditions. These can be considered equations of choice for the specific age groups, disease conditions, and ethnicities within Asia.

## 1. Introduction

Accurately estimating renal function plays a critical role in diagnosing, staging, and managing chronic kidney disease (CKD) [1]. Glomerular filtration rate (GFR) has gained the status of indicator of choice for assessment of renal function whereas clearance of inulin administered by infusion and its sampling in urine has become a “gold standard” for measuring GFR [2]. Nowadays, the plasma clearance of other exogenous filtration markers e.g., 99mTc-DTPA has been approved to exhibit GFR adequately [3,4] They are easier to perform in comparison to inulin but still, it is laborious work to do. The current estimation of GFR takes into account the individual’s race, sex, age, and weight as non-determinants of GFR for serum creatinine (Scr) [5,6,7], as the production of Scr is proportional to these variables as well as to the muscle mass [8]. However, the utilization of Scr for the estimation of GFR is not a method of choice when the level of renal impairment reaches 50% or less [9]. Widely employed creatinine-based equations in clinical practice include Cockcroft-Gault (CG) [6], Modification of Diet in Renal Disease (MDRD) [10], and creatinine-based Chronic Kidney Disease Epidemiology Collaboration (CKD-EPI-Cr) equations [5]. Equations based on standardized cystatin C (CKD-EPI cystatin C) or cystatin C and creatinine (combined CKD-EPI equation) have also been proposed by the CKD-EPI consortium in 2012 [11]. More recently, the New European Kidney Function Consortium (EKFC) equation has been developed which is a modified FAS SCr-based equation combining the design feature of FAS and CKD-EPI equations [12]. In addition, the National Kidney Foundation and the American Society of Nephrology recommend immediately replacing older eGFRcr equations (MDRD Study and CKD-EPI 2009) with the new 2021 CKD-EPI equation [13,14].

Several equations have been evolved so far that employ serum cystatin C, a newer biomarker for GFR estimation, either alone or in combination with serum creatinine. Cystatin C equations involve fewer covariates as it does not depend on such parameters as height, weight, age, and sex, which are often part of creatinine-based estimation equations. A cystatin C-based equation developed by Hoek et al. exhibited a higher precision and accuracy in comparison to the Cockroft-Gault equation [15]. Over many years, several other cystatin C-based equations for eGFR have evolved, such as Flodin [16], Grubb [17], and Larsson [18] equations. When compared with creatinine-based eGFR equations, these equations were beneficial for early diagnosis of renal dysfunction and it is considered a risk predictor of higher quality among patients suffering from diabetes mellitus [19]. Other cystatin C-based equations include CAPA [20] and full-Age Spectrum (FAS) equation [21].

There exists a significant gap between cystatin C and creatinine-based estimation of GFR when using the CKD-EPI equation. Several clinical trials have conducted the comparison of creatinine and cystatin C in CKD-EPI equations for estimating GFR, but the results have remained a matter of argument. It has been recommended in The Kidney Disease Improving Global Outcome (KDIGO) guidelines (2012) to use the CKD-EPI-Cr-CysC among adults with eGFR in between the range of 45–59 mL/min/1.73 m^2^ with no signs or markers of renal damage [22]. However, employing cystatin C is linked with higher medical costs [23]. Reagents for cystatin C are more expensive than for serum creatinine (around $4 versus $0.20 per test) [24]. 

Thus far, in numerous studies, creatinine-based equations have been modified by the addition of an ethnicity coefficient for Asians, for example, Chinese [25]. Cystatin C-based equations have not been practiced extensively in the Asian population. Despite the ascending research on eGFR equations all around the world, there is little known about the best equations in Asia and there is a scarcity of reviews that call attention to the optimal ones. In addition, there are a large number of equations developed so far and it has become difficult for clinical researchers, nephrologists, and other clinicians to pick out the best available equation in a given age group, disease condition, and ethnicity. This review has been undertaken by us to gather evidence for the optimal GFR equations in Asian populations for various age groups and disease conditions, and to see if the equations based on a combination of creatinine and cystatin C biomarkers, if employed, are superior in performance compared to those based on a single biomarker in different age groups, disease conditions and across various ethnicities and populations in Asia. 

## 2. Methods

### 2.1. Protocol

The systematic review’s protocol has been developed according to Preferred Reporting Items for Systematic Reviews and Meta-analyses (PRISMA) [26] guidelines and registered in PROSPERO (International Prospective Register of Systematic Reviews; https://www.crd.york.ac.uk/prospero, registration number: CRD42021276236). However, a meta-analysis was not carried out and 14 elements of the PRISMA checklist are marked as “N/A”. Performing a meta-analysis was not feasible as most of the studies included were clinically diverse and had a high risk of bias which will produce misleading results (“See Supplementary Data Table S1”).

### 2.2. Eligibility Criteria

We included validation studies that had both creatinine and cystatin C-based equations, either alone or in combination, to estimate GFR in any Asian populations, where validated samples with specific disease conditions, had P30 values of accuracy, and those which compared the accuracy of these equations with measured GFR (mGFR) by exogenous markers (99mTc-DTPA, 51Cr-EDTA, Inulin, and Iohexol) as the gold standard. 

The exclusion criteria were the absence of accurate results to evaluate the performance of equations, studies carried out in the general population without specific disease conditions, use of urinary cystatin C or creatinine instead of serum cystatin C or creatinine as GFR marker, review studies, and animal studies. Original validation studies written only in English were considered.

### 2.3. Search Strategy and Study Selection

A systematic search of Pubmed, EMBASE, Cochrane Library, and Web of Science was conducted from 2012 to 2020 by two independent reviewers. The search strategy using a wide range of Medical Subject Headings (MeSH) was as follows: “(“Creatinine and Cystatin C”[Mesh] OR “Glomerular filtration rate”[Mesh] OR “Asian population”[Mesh]) AND (“Validation”[Majr] or “Creatinine”[Majr] OR “Cystatin C”[Majr] OR “Validation”[Majr] OR “eGFR equations”[Mesh]) AND (“Cr” [Mesh] OR “CysC”[Mesh] AND Cystatin C”[Mesh] AND “Asian”[Mesh])”. 

All records were assessed by independent reviewers based on titles and abstracts for inclusion in this review. Abstracts that do not match the inclusion criteria or those that matched the exclusion criteria were discarded. The records that remained and those with abstracts providing adequate information for deciding upon their inclusion were considered for full-text evaluation which was performed independently by the same reviewers. Disagreements were solved by a third reviewer. 

### 2.4. Data Collection and Extraction

The selected studies were analyzed by two investigators and the data were extracted by utilizing the standardized system accordingly. The following information was gathered: first author, publication year, sample size, measured GFR (mGFR) value, population, age group, and disease condition in which equations were validated, evaluated creatinine and cystatin C- based equations, reference method for mGFR, creatinine, and cystatin C measurement methods, and whether or not they were traceable to the reference method, bias, precision, and 30% accuracy (P30) of the equation with measured GFR. The populations in the selected studies were categorized according to age groups and specific disease conditions. Age groups were defined on the basis of age ranges; Children + Adolescents = ≤18 years, Adults = 18–60 years, and Elderly = ≥60 years. The optimal equations for each category were then identified on the basis of pre-specified criteria given in KDIGO guidelines (bias, precision, and P30) [27]. Finally, optimal equations were suggested for clinical application. In order to assess the clinical applicability of each suggested equation, disease conditions, age groups, and ethnicities were predicted on the basis of validation studies whereas the clinical settings in which an equation can be applied were approximated on the basis of the study population from which individual equation was derived. 

### 2.5. Risk of Bias in Individual Studies and Quality of Systematic Review 

The quality and risk of bias of studies were evaluated with the Quality of Diagnostic Accuracy Studies-2 (QUADAS-2) tool [28].

### 2.6. Diagnostic Accuracy Measures and Selection of Optimal Equations

Following the guidelines of KDIGO, accuracy was defined as P30, the percentage of eGFR values within 30% of measured GFR. The bias (mean or median difference) and precision (standard deviation, SD; or interquartile range, IQR of difference) among eGFR and mGFR were recorded accordingly. Optimal equations were selected on the basis of these diagnostic accuracy measures outlined by KDIGO guidelines [27]. 

## 3. Results

**Figure 1:**
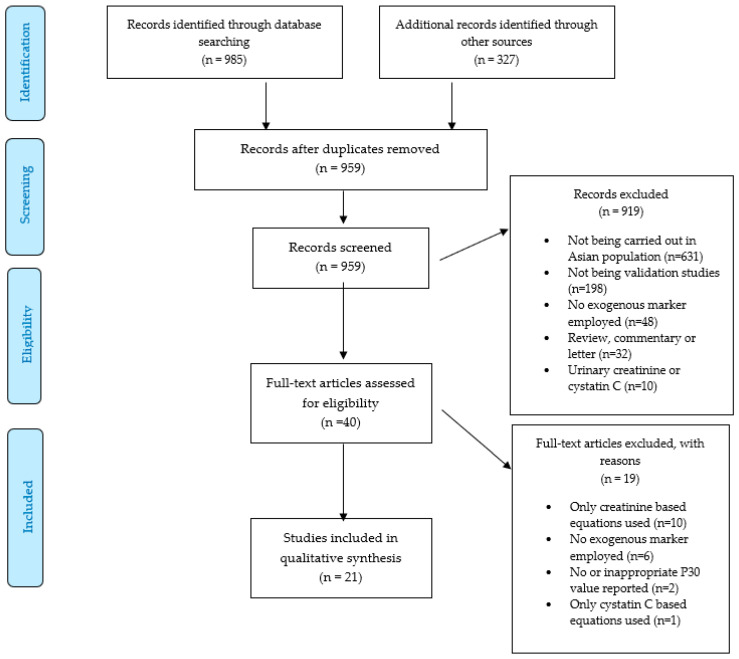
PRISMA Flow chart.

Our search strategy identified 1312 citations, out of which 959 were left after removing 353 duplicates and unrelated papers. We excluded 919 articles after the screening of titles and abstracts, after which 40 studies were left for full-text evaluation. There were 19 [2,8,29,30,31,32,33,34,35,36,37,38,39,40,41,42,43,44,45] full-text articles that were excluded. Finally, 21 studies [9,46,47,48,49,50,51,52,53,54,55,56,57,58,59,60,61,62,63,64,65], which included 11,371 participants consisting of a broad range of values for GFR, were chosen for systematic review. Figure 1 represents the PRISMA flowchart for study selection.

Finally, the overall quality of included studies were assessed by the QUADAS-2 tool (“See Supplementary Data Table S1”). The studies were selected in the time range of 2012 and 2020 and 54 equations were extracted (“See Supplementary Data Table S2”) which were either derived from creatinine or cystatin-C alone or a combination of them. The main characteristics of studies with best-performed equations are shown in Table 1. 

The populations in the selected studies were categorized according to age groups, specific disease conditions, and ethnicities: Four of them were performed on elderly CKD patients (Four Chinese [48,50,52,54]), eleven in adult and elderly CKD patients (eight Chinese [9,46,55,59,60,62,63,64], one Japanese [51], one Indian [53], and one multiethnic study [57]), and two in adult renal transplant recipients (one Chinese [56], and one Korean study [61]). Whereas only single studies were found among Chinese children and adolescents with renal injury [65], Chinese adult patients with obstructive nephropathy [49], Japanese adult cirrhotic patients [47], and Chinese adult CKD diabetic and non-diabetic patients [58]. Among the methods used for measurement of serum biomarkers, eleven studies reported the use of immunoturbidimetry, seven studies used immunonephelometry, one used latex enhanced immunoturbidimetric, one used colloidal gold immunoassay and one did not report the method used. The bias (mean or median difference), precision (SD or IQR of difference,) and P30 accuracies of the equations ranged from −14.54 to 9.96, 1.61 to 59.85, and 4.7 to 96.10. The highest values of P30 accuracies were found for the JSN-CKDI equation (96.10%) in Chinese adult renal transplant recipients, for the BIS-2 equation (94.5%) in Chinese elderly CKD patients, and Filler equations (93.70%) also in Chinese adult renal transplant recipients. Optimal equations in various ethnicities across different age groups and disease conditions are illustrated in Table 2 Additionally, the optimal equations have also been suggested along with their clinical applicability in Table 3. 

### 3.1. Optimal Equations in Different Age Groups, Specific Disease Conditions, and Ethnicities

#### 3.1.1. Optimal Equations for Elderly CKD Population

As far as the performance of various equations in Chinese elderly CKD patients is concerned, Guan Changjie et al. demonstrated that a creatinine and cystatin C combination equation, BIS-2 (Berlin Initiative Study) has a better performance compared to MDRD and CKD-EPI equations in estimating glomerular filtration in elderly CKD patients [48]. Similar findings were observed in another validation study performed by them which included 368 elderly CKD patients which also supported the optimal performance of the BIS-2 equation at GFR 30 mL/min/1.73 m^2^ or greater in comparison to Feng-Cr-CysC, CKD-EPI-Cr-CysC, and MA-Cr-CysC equations. However, the CKD-EPI-Cr-CysC equation yielded better performance in patients with measured GFR less than 30 mL/min/1.73 m^2^ [50]. 

Similarly, Huang et al. screened different equations which have previously shown high accuracy among the Chinese elderly population. In this study, CG (at mGFR ≤ 60 mL/min/1.73 m^2^), Modified CKD-EPI-Cr-CysC for elderly (at mGFR ≥ 60 mL/min/1.73 m^2^), Standardized SCr and SCysC CKD-EPI (at mGFR ≥ 60 mL/min/1.73 m^2^) showed optimal performance [52]. On the other hand, modified CKD-EPI-CysC for the elderly and modified CKD-EPI-Cr-CysC for the elderly by Fen li et al., were validated in Chinese which proved the higher accuracy of the modified equations than the original one in the elderly CKD population [54]. So, these identified optimal equations perform best in the Chinese elderly CKD population and should be further validated in other Asian countries and ethnicities in elderly CKD patients. 

#### 3.1.2. Optimal Equations for Both Adult and Elderly CKD Population

Min Yang et al. performed a validation study on 632 adult and elderly Chinese CKD patients in which CKD-EPI equations were validated. This study demonstrated the reliability of CKD-EPI-Cr-CysC and CKD-EPI-CysC for assessing CKD stages correctly in these individuals. CKD-EPI-Cr-CysC equation has shown particularly better accuracy and diagnostic value in participants with normal or mildly impaired GFR, while the CKD-EPI-CysC equation performed better in CKD stages 3–4 [59]. These results were consistent with Hua Chi et al. study which showed that the employment of a combination of cystatin C and serum creatinine (CKD-EPI-Cr-CysC) levels show improvement in the bias of equation and achieves greater diagnostic accuracy in patients with renal insufficiency [46]. Another validation study was performed by Kumar et al., where the cystatin-C-based equation showed superior performance in the adult CKD population. Stratification by measured GFR and by gender or age did not change the results. This equation showed P30 values of 81.5% and 69.7% for those having measured GFR ≥60 mL/min/1.73 m^2^ and <60 mL/min/1.73 m^2^, respectively [53].

In addition, two prospective cohorts were considered from the Chinese population, and three new equations C-CKD-EPI-Cr, C-CKD-EPI-CysC, and C-CKD-EPI-Cr-CysC were developed in one and validated in the other. The best accuracy with the highest P30 value was depicted by C-CKD-EPI-CysC with relatively lower precision but C-CKD-EPI-Cr-CysC depicted improved accuracy, bias, and precision [60]. C-CKD-EPI-Cr-CysC had also shown the lowest bias in CKD stage three to five among adults and elderly in another validation study by Yue L. et al. [64]. This study also showed optimal performance of two other equations; Xiangya and CKD-EPI-CysC. Feng et al. [9] and Horio et al. [51] also validated eGFR equations in the CKD population and found that Feng-Cr-CysC, Feng-CysC, and CKD-EPI-CysC were ideal equations. In addition, Teo BW et al. [57] performed validation in a multiethnic Asian population with CKD and found that standardized SCr and SCysC CKD-EPI is the best-performed equation for the multiethnic Asian population. 

The eGFR equations have also been validated by Xiaoshuang Ye et al. in the Asian population [62]. Among all equations, the Feng-Cr-CysC equation achieved the best performance, although still requires improvement when applying to GFR less than 60 mL/min per 1.73 m^2^ in clinical settings. A favorable performance was achieved by the Feng-CysC equation too, which was only less efficient than the Feng-Cr-CysC equation On the other hand, previous studies in Asia have demonstrated the inaccuracy of CKD-EPI-Cr-CysC and CKD-EPI-CysC among aging cohorts with GFR at a moderately severe level of impairment. Thus, the assessment of adaptability and performance of the new FAS equation was assessed in a validation study performed by Yong et al. [63] which proved that the FAS-Cr-CysC equation has superior diagnostic accuracy among whole subjects (in the subgroup rGFR less than 60 mL/min per 1.73 m^2^), particularly in elderly patients with GFR at a moderately severe level of impairment. Another validation study performed by Pei et al. [55] proved the accuracy of modified MDRD by Pei, modified CKD-EPI by Pei, and Pei (Modified Maclssac) in the adult and elderly Chinese CKD population. 

Hence, these identified optimal equations are ideal for implementation in adult and elderly CKD patients in their respective ethnicities in which they have been validated. In addition, these identified equations can be studied in the future and validated in other countries and ethnicities within Asia in CKD patients.

#### 3.1.3. Optimal Equations for Obstructive Nephropathy and Kidney Transplant Recipients

A validation study performed by Chen et al. [49] in Chinese has proved that the CKD-EPI-Cr-CysC equation showed the best performance in obstructive nephropathy patients too. CKD-EPI-CysC equation showed high accuracy and lesser bias in individuals with the lowest GFR. In patients with high GFR, CKD-EPI-Cr-CysC was the best in performance, although both CKD-EPI-CysC and CKD-EPI-Cr-CysC had a huge bias in these patients. In addition, the bias of all the equations was larger in women as compared to men. However, CKD-EPI-Cr-CysC had the highest accuracy for both males and females. 

A validation study performed by Yang et al. [61] in the Korean population exhibited better performance of cystatin C equations in kidney transplant recipients with lesser GFR. CKD-EPI-CysC exhibited the least bias and highest precision in patients with measured GFR less than 45 mL/min/1.73 m^2^ and all other equations underestimated measured GFR significantly. 

In another study performed by Tang et al., [56] in Chinese kidney transplant recipients, JSN-CKDI, Larsson, Rule, 2003 Hoek, Filler, and Grubb equations exhibited the best performances. In this study, it was observed that almost all the equations have elevated net biases when the GFR value was greater than 60 mL/min/1.73 m^2^. It has also been indicated that GFR equations are unsuitable for renal transplant recipients whose GFR values are at a high level. 

#### 3.1.4. Optimal Equations for Diabetic CKD Population

It has always been controversial to select an optimal equation for the estimation of GFR in CKD patients with diabetes. Hence, the lower accuracy of GFR estimating equations in diabetic CKD patients led to another Chinese validation study performed by Xie et al. [58] on 215 diabetic CKD patients and 192 non-diabetic CKD patients. Here too, CKD-EPI-Cr-CysC exhibited the best performance among all CKD-EPI equations. However, BMI, CKD status, mGFR, and HbA1c are considered independent factors which are associated with the accuracy of eGFR equations. 

#### 3.1.5. Optimal Equations for Renal Injury Children

The glomerular filtration rate is vital for the evaluation of renal function and classification of CKD in children, while the reference technique in children is inconvenient [66]. In Chinese children, the non-availability of data about GFR measurement by renal or plasma clearance of exogenous markers has led to the unavailability of validated estimating tools for GFR in this population. A validation study carried out by Zheng et al. [65] in this age group showed an accurate estimation of GFR by cystatin-C-based CKiD and Filler equations. These equations can be employed as equations of choice in Chinese renal injury children and can further be validated in renal injury children in other populations and ethnicities within Asia. 

#### 3.1.6. Optimal Equation for Liver Cirrhosis Population

The assessment of kidney function is of significant importance in managing patients with cirrhosis. Creatinine-based estimating equations do not depict the true renal function because of impairment of liver function and muscle wasting, although serum creatinine is used routinely for this purpose. Cystatin C, by contrast, is not related to liver function and muscle volume. In a Japanese validation study performed by Adachi et al. [47] cystatin C- based equations were examined for assessing the renal function in Japanese cirrhotic patients. The outcome suggested that the eGFR-CysC equation estimated the renal function and predicted the results in a more accurate way as compared to Cr-based estimating equations in patients with cirrhosis. Hence, eGFR-CysC can be considered best while evaluating kidney function in the cirrhotic population. 

## 4. Discussions

Estimated glomerular filtration rate equations, either alone or with a combination biomarker, in the selected studies, were validated in different ethnicities including Chinese, Japanese, Korean, Malay, and Indian populations. Our review assists clinical researchers, nephrologists, and other clinicians who can apply the identified optimal equations (Table 3) in routine practice to make accurate treatment decisions relying upon the GFR estimation in the Asian population in the respective age groups, ethnicities, and disease conditions. The results of our review emphasize the use of cystatin C and creatinine combination equations for estimating GFR in a multiethnic Asian population in most of the age groups and disease conditions which include chronic kidney disease, diabetes, renal injuries, obstructive nephropathy, renal transplant, and liver cirrhosis (Table 2). Although equations based only on cystatin C have also shown ideal performances in some of the included studies (Table 2). 

Previous studies have also implied the fact that serum creatinine-based equations underestimate and serum cystatin C-based equations overestimate GFR, and the average yield the best estimate [67,68]. Our review has depicted similar findings across different age groups, disease conditions, and ethnicities in Asia. This may be related to factors responsible for the generation of these biomarkers. Serum creatinine generation reflects the amount of lean muscle mass [69]. Body mass index influences serum cystatin C generation [70]. It has been suspected that the degree of ethnic and environmental influences on fat and muscle proportion is rendered less important once the GFR estimates are averaged using a combination of these two markers [57]. Thus, our review also supports the idea that ethnicity adjustment is not required for the combination biomarker equation in multiethnic Asian populations. However, the implementation of cystatin C estimation is difficult in developing countries of Asia because of its associated cost especially for monitoring CKD without any improvement in risk prediction [71] as well as standardization issues for calibration. This has led to the limited use of cystatin C-based equations in the Asian population and steps needs to be taken to generalize the utilization of such useful biomarker in Asian laboratories. 

Some limitations of our review must be taken into account. First, there is a lack of reliable methods for assessment of the risk of publication bias in such systematic reviews as ours which are based on observational studies. Despite the thorough research of the literature, the likelihood of such bias in the present review is not known. Secondly, most of the studies (77%) included in this systematic review came out from the Chinese population. There is a lack of potential validation studies being carried out in other Asian countries for eGFR equations. Furthermore, there is a rigorous need for external validation studies for eGFR equations in Asian countries. Moreover, data is confined to the Asian population only. Thirdly, the results of this review could have high statistical and clinical heterogeneity as the comparisons have limitations in the way in which the validation studies were carried out and the methodology of data reporting in these studies. Moreover, the participants were not recruited from community settings in the majority of included studies. Another limitation of our review includes the use of DTPA scintigraphy (Gates method) in the studies included, although this method is not a reference method as it cannot provide convincing data to support the preferred use of any equation as this method is affected by many factors other than GFR itself and can only be used to determine relative GFR [72]. Additionally, the 99mTc-DTPA was utilized as the reference method for mGFR in almost 80% of studies included in this study. Studies have shown that this method is not suitable for the gold standard for mGFR measurement [73,74,75]. Therefore, errors might exist in the equation evaluations. 

## 5. Conclusions

Finally, three equations [BIS-2, CG, modified equations for elderly] are ideal for elderly CKD individuals whereas four equations [(Standardized SCr & SCysC CKD-EPI), (CKD-EPI-Cr-CysC & CKD-EPI-CysC), (C-CKD-EPI-Cr-CysC & C-CKD-EPI-CysC), (FAS-Cr-CysC)] are excellent to be applied in adult CKD individuals. Furthermore, the Filler equation is optimal in renal injury for both children and adults, whereas the eGFR-CysC best fitting equation in liver cirrhosis. For renal transplants, JSN-CKDI and Rule equations stand out among other equations. 

This systematic review has summarized and suggested the optimal GFR equations for various age groups and disease conditions in Asian populations. This review has illustrated that cystatin C and creatinine combination equations are more precise and accurate in most of the age groups, disease conditions, and ethnicities than equations that are based on either of the biomarkers. Although equations based on cystatin C alone were also ideal in certain age groups and disease conditions. Nephrologists, clinical researchers, and other clinicians can apply suggested equations for accurate decision making and suitable patient outcomes can be achieved in the respective age group, disease condition, and ethnicity in which they have been identified. There is a huge heterogeneity within the continent. Hence, in order to support the findings of this review, further validation studies are required for these optimal equations within other ethnicities and races in Asia in their respective age groups and disease conditions in which they have been identified.

## Figures and Tables

**Table 1:** Characteristics of included studies.

Author, Year, Country	Population	Reference Standard	mGFR (ml/min/1.73 m^2^)	Optimal Equations
Xiaoshuang Ye, 2016, China [62]	Chinese adult patients n= 1522	99mTc-DTPA	67.30 ± 28.89	Feng-Cr-CysC
Min Yang, 2017, China [59]	Chinese CKD patients n = 632	99mTc-DTPA	64.82 ± 31.97	CKD-EPI-Cr-CysC
Guan Changjie, 2017, China [48]	Chinese elderly CKD patients n = 218	99mTc-DTPA	47.62 (3.00–135.00)	BIS-2
Zheng, 2017, China [65]	Chinese renal injury children n = 87	99mTc-DTPA	97.0 ± 31.9	CkiD and Filler
Kumar, 2018, India [53]	Indian CKD patients n = 67 Healthy donor n = 63	Inulin	51.66 ± 31.68	CKD-EPI-CysC
Tang, 2018, China [56]	Chinese renal transplant recipients n = 252	99mTc-DTPA	66.2 ± 19.1 (16.1–126.7)	JSN-CKDI Larsson Rule 2003 Hoek Filler Grubb
Yang, 2017, Korea [61]	Korean renal transplant recipients n = 70	99mTc-DTPA	57.6 ± 16.5	MDRD CKD-EPI-CysC
Chen, 2016, China [49]	Chinese adult patients with obstructive nephrology n = 245	99mTc-DTPA	66.54 ± 23.99	CKD-EPI-Cr-CysC
Guan Changjie, 2018, China [50]	Chinese elderly CKD patients n = 368	99mTc-DTPA	47.1 (1.0–12/9.2)	CKD-EPI-Cr-CysC BIS-2
Huang, 2015, China [52]	Chinese elderly CKD patients n = 151 Healthy individuals n = 94	99mTc-DTPA	65.39 ± 24.19	CG Modified CKD-EPI-Cr-CysC for elderly Standardized SCr and SCysC CKD-EPI
Yang, 2019, China [60]	Chinese CKD patients n = 842 Development group n = 529 Validation group n = 313	99mTc-DTPA	47(9.9–107.5)	C-CKD-EPI-CysC
Xie, 2019, China [58]	Chinese diabetic CKD patients n = 215 Chinese non-diabetic CKD patients n = 192	Iohexol	50.30 (31.43)	CKD-EPI-Cr-CysC
Adachi, 2015, Japan [47]	Japanese cirrhotic patients n = 63 First study n = 14 Follow-up study n = 49	Inulin	54.4 (38.9–97.1)	eGFR-CysC
Yong, 2018, China [63]	Chinese patients n = 1184	99mTc-DTPA	67.33 (41.37, 87.50)	FAS-Cr-CysC
Hua Chi, 2017, China [46]	Chinese adult CKD patients n = 1296	99mTc-DTPA	46.8 (29.8, 68.3)	CKD-EPI-Cr-CysC
Fen Li, 2016, China [54]	Chinese elderly patients n = 839 Training set n = 674 Verification set n = 165	99mTc-DTPA	51.88 ± 22.60	Modified CKD-EPI-CysC for elderly Modified CKD-EPI-Cr-CysC for elderly
Yue l., 2020, China [64]	Chinese CKD patients n = 830	99mTc-DTPA	55.2 (10–108.1)	CKD-EPI-CysC C-CKD-EPI-Cr-CysC Xiangya
Pei, 2013, China [55]	Chinese adult patients n = 703	99mTc-DTPA	77.14 ± 25.93	Modified MDRD by Pei Modified CKD-EPI by Pei Pei (Modified Maclssac)
Feng, 2013, China [9]	Chinese CKD patients n = 788	99mTc-DTPA	50.84 ± 31.36	Feng-Cr-CysC Feng-CysC
Horio, 2013, Japan [51]	Japanese CKD patients n = 763	Inulin	57.20 ± 34.7	CKD-EPI-CysC
Teo BW, 2012, Singapore [57]	Multiethnic CKD patients n = 232	99mTc-DTPA	51.70 ± 27.50	Standardized SCr and SCysC CKD-EPI

Abbreviations: CKD, chronic kidney disease; CysC, cystatin C based equation; Cr-CysC, equation based on both creatinine and cystatin-C suggested by the kidney Disease: Improving Global Outcomes Guidelines; CKD-EPI, Chronic Kidney Disease Epidemiology Collaboration; eGFR-CysC, estimated glomerular filtration rate based on cystatin-C; C-CKD-EPI, modified CKD-EPI equation for the Chinese population; BIS, Berlin Initiative Study.

**Table 2:** Optimal Equations in various ethnicities across different age groups and disease conditions.

Disease Condition	Age Group	Studies	Ethnicity	Optimal Equations
CKD Population	Elder	Guan Changjie, 2017, China [48]	Chinese	BIS-2
		Guan Changjie, 2018, China [50]	Chinese	BIS-2 CKD-EPI-Cr-CysC
		Huang, 2015, China [52]	Chinese	CG (mGFR ≤ 60 mL/min/1.73 m^2^) Modified CKD-EPI-Cr-CysC for elderly (mGFR ≥ 60 mL/min/1.73 m^2^) Standardized SCr and SCysC CKD-EPI (mGFR ≥ 60 mL/min/1.73 m^2^)
		Fen Li, 2016, China [54]	Chinese	Modified CKD-EPI-CysC for elderly Modified CKD-EPI-Cr-CysC for elderly
CKD Population	Adult + Elderly	Feng, 2013, China [9]	Chinese	Feng-Cr-CysC Feng-CysC
		Horio, 2013, Japan [51]	Japanese	CKD-EPI-CysC
		Teo BW, 2012, Singapore [57]	Multiethnic (Chinese, Malay, Indian, and others)	Standardized SCr and SCysC CKD-EPI
		Min Yang, 2017, China [59]	Chinese	CKD-EPI-Cr-CysC
		Yang, 2019, China [60]	Chinese	C-CKD-EPI-CysC
		Hua Chi, 2017, China [46]	Chinese	CKD-EPI-Cr-CysC
		Yue l., 2020, China [64]	Chinese	CKD-EPI-CysC C-CKD-EPI-Cr-CysC Xiangya
		Xiaoshuang Ye, 2016, China [62]	Chinese	Feng-Cr-CysC
		Yong, 2018, China [63]	Chinese	FAS-Cr-CysC (particularly in elder patients)
		Pei, 2013, China [55]	Chinese	Modified MDRD by Pei Modified CKD-EPI by Pei Pei (Modified Maclssac)
		Kumar, 2018, India [53]	Indian	CKD-EPI-CysC
Renal Injury Population	Children + Adolescent	Zheng, 2017, China [65]	Chinese	CKiD Filler
Diabetic and Non-diabetic CKD Population	Adult	Xie, 2019, China [58]	Chinese	CKD-EPI-Cr-CysC
Population with Liver Cirrhosis	Adult	Adachi, 2015, Japan [47]	Japanese	eGFR-CysC
Population with Obstructive nephropathy/Renal Transplant	Adult	Chen, 2016, China [49]	Chinese	CKD-EPI-Cr-CysC
		Tang, 2018, China [56]	Chinese	JSN-CKDI Larsson Rule 2003 Hoek Filler Grubb
		Yang, 2017, Korea [61]	Korean	MDRD CKD-EPI-CysC (mGFR ≤ 45 mL/min/1.73 m^2^)

Abbreviations: CKD, chronic kidney disease; CysC, cystatin C based equation; Cr-CysC, equation based on both creatinine and cystatin-C suggested by the kidney Disease: Improving Global Outcomes Guidelines; CKD-EPI, Chronic Kidney Disease Epidemiology Collaboration; eGFR-CysC, estimated glomerular filtration rate based on cystatin-C; C-CKD-EPI, modified CKD-EPI equation for the Chinese population; BIS, Berlin Initiative Study.

**Table 3:** Suggested Equations and their Clinical Applicability.

Suggested Equation	Clinical Applicability of Suggested Equations in Asian Population					
	Disease Condition	Age Group	Ethnicity	Clinical Settings in Which the Equation Can Be Applied	Comments	Limitations in Studies from Which Equations Were Derived
BIS-2	CKD	Elderly	Chinese	Outpatient, Population screenings	Ideal for estimating GFR in ≥70 years with normal or mild to moderately reduced kidney function	No randomization of participants No external validation set
CG	CKD	Elderly	Chinese	Inpatient	Useful for quickly predicting creatinine clearance without collecting urine	Several limitations in the prediction of creatinine clearance and serum creatinine
Modified CKD-EPI-Cr-CysC for the Elderly Modified CKD-EPI-CysC for the Elderly	CKD	Elderly	Chinese	Outpatient, Population screenings, Inpatient	These modified equations are more accurate than the original ones for elderly Chinese individuals	Limited external validation
Standardized SCr & SCysC CKD-EPI	CKD	Adult Elderly	Multiethnic (Chinese, Malay, Indian, and others)	Population screenings	Best for population screenings but can also be employed at bedside and outpatient settings after validation	Only one external validation data set was used Pooled analysis, rather than from a representative population
CKD-EPI-Cr-CysC CKD-EPI-CysC	CKD, obstructive nephropathy, and renal transplant	Adult Elderly	Chinese Japanese Indian Korean	Outpatient, Population screenings, Inpatient	Although included a large patient population during development but still needs further validation in Asia Widely accepted alternative in both elders and adults with normal to mildly injured kidneys	No racial or ethnic minorities Incomplete data on muscle mass & other clinical conditions Errors in mGFR
C-CKD-EPI-Cr-CysC C-CKD-EPI-CysC	CKD	Adult Elderly	Chinese	Outpatient, Population screenings, Inpatient	Improved efficacy in Chinese population & can be applied to other ethnicities in Asia after validation	Small validation set
FAS-Cr-CysC	CKD	Children Adolescents Adult Elderly	Chinese	Outpatient, Population screenings, Inpatient	Accurate in sub-group ≤ 60 mL/min/1.73 m^2^ particularly in elderly	Were limited to Caucasians during development so still needs extensive external validation
Filler	Renal Injury	Children Adolescents Adults	Chinese	Outpatient, Population screenings	This equation can effectively replace Schwartz equation	Large variability of serum creatinine & cystatin C
eGFR-CysC	Liver Cirrhosis	Adult	Japanese	Outpatient, Population screenings, Inpatient	Validated in the Japanese population with liver cirrhosis but require validation in other ethnicities with this disease within Asia	No diverse developmental data set
JSN-CKDI	Renal Transplant	Adult	Japanese Chinese	Inpatient	More studies are required to check the accuracy of this equation in other regions of Asia	Possible variation in creatinine assays overtime Exclusion of patients with higher GFR
Rule	Renal Transplant	Adult	Chinese	Outpatient, Population screenings	Depending on the clinical settings, this equation can be averaged with SCr equation or used in place of it	Generalizability needs to be tested in more diverse ethnic groups

Abbreviations: CKD, chronic kidney disease; CysC, cystatin C based equation; Cr-CysC, equation based on both creatinine and cystatin-C suggested by the kidney Disease: Improving Global Outcomes Guidelines; CKD-EPI, Chronic Kidney Disease Epidemiology Collaboration; eGFR-CysC, estimated glomerular filtration rate based on cystatin-C; C-CKD-EPI, modified CKD-EPI equation for the Chinese population; BIS, Berlin Initiative Study.

## Data Availability

The data presented in this study are available in supplementary material.

## Supplementary Materials

The following supporting information can be downloaded at: https://www.mdpi.com/article/10.3390/jcm12051822/s1, Table S1: Quality assessment of included studies using QUADAS-2; Table S2: List of extracted equations with their abbreviations, expressions and references. References [76,77,78,79,80,81,82,83,84,85,86,87,88,89,90,91,92,93,94] are cited in the supplementary materials.
