# Supplementary material for: Optimal Glomerular Filtration Rate Equations for Various Age Groups, Disease Conditions and Ethnicities in Asia: A Systematic Review
Source: J Clin Med. 2023 Feb 24;12(5):1822. doi: 10.3390/jcm12051822 (PMC10002889; doi:10.3390/jcm12051822)
Supplement: Supplementary file 1 [file jcm-12-01822-s001.zip › Supplementary Data Table S1.pdf]

Supplementary Data Table S1

Table S1 Quality assessment of included studies using QUADAS-2

Study	Risk of bias				Applicability concerns		
	Patient selection	Index test	Reference test	Flow and timing	Patient selection	Index test	Reference test
Xiaoshuang [1]	High	Low	High	Low	Low	Low	Low
Yong [2]	High	Low	High	Low	Low	Low	Low
Min-Yang [3]	High	Low	High	Low	Low	Low	Low
Zheng [4]	High	Low	High	Low	Low	Low	Low
Kumar [5]	Low	Low	Low	Low	Low	Low	Low
Tang [6]	High	Low	High	Low	Low	Low	Low
Yang 2017 [7]	Low	High	High	Low	Low	Low	Low
Chen [8]	Low	Low	High	Not clear	Low	Low	Low
Guan 2017 [9]	Low	Low	High	Not Clear	Low	Low	Low
Guan 2018 [10]	Low	Low	High	Low	Low	Low	Low
Huang [11]	Low	Low	High	Not clear	Low	Low	Low
Can Hu [12]	Low	Low	High	Not clear	Low	Low	Low
Yang 2019 [13]	Low	Low	High	Not clear	Low	Low	Low
Xie [14]	Low	Low	Low	Not clear	Low	Low	Low
Adachi [15]	Low	Low	Low	Low	Low	Low	Low
Fen li [16]	Low	Low	High	Low	Low	Low	Low
Yue L. [17]	Low	Low	High	Low	Low	Low	Low
Pei [18]	Low	Low	High	Low	Low	Low	Low
Feng [19]	Low	Low	High	Low	Low	Low	Low
Horio [20]	Low	Low	Low	Low	Low	Low	Low
Teo BW [21]	Low	Low	High	Low	Low	Low	Low

For the "Patient Selection" domain, the risk of bias was variable across the studies selected, as the description of processes involved in recruitment were either inconsistent or a group of healthy controls were recruited with the group having target clinical condition. For domains of "index test" and "reference test" there was an unclear risk of bias in these studies as they did not describe whether results of mGFR and eGFR were interpreted without knowledge of each other. For the "index test" domain only the study conducted in the Korean population with renal transplant recipients [1] had a high risk of bias as the thyroid function test was not performed after 1 year of kidney transplantation which can affect the interpretation of serum cystatin C test and might have introduced bias. For "reference test" we concluded that 17 studies (80%) ([1], [2], [3], [4], [6], [7], [8], [9], [10], [11], [12], [13], [16], [17], [18], [19], [21]) have high risk of bias, as 99mTc-DTPA has been shown to be unsuitable to be employed as reference method especially when investigating CKD-EPI equation validity and interfere with interpretation of results. For the domain of "flow and timing" we observed that 5 (23.8%) studies ([8], [9], [11], [12], [13]) have an unclear risk of bias as they did not report the interval between the index test and reference test. The applicability concerns were low in the included studies.

References:

1. Ye X, Liu X, Song D, Zhang X, Zhu B, Wei L, et al. Estimating glomerular filtration rate by serum creatinine or/and cystatin C equations: An analysis of multi-centre Chinese subjects. Nephrology 2016;21: 372-378.
2. Yong Z, Li F, Pei X, Liu X, Song D, Zhang X, et al. A comparison between 2017 FAS and 2012 CKD-EPI equations: a multi-center validation study in Chinese adult population. *Int Urol Nephrol* 2019;51: 139-146.
3. Yang M, Xu G, Ling L, Niu J, Lu T, Du X, et al. Performance of the creatinine and cystatin C-based equations for estimation of GFR in Chinese patients with chronic kidney disease. *Clin. Exp. Nephrol* 2017;21:236-246.
4. Zheng K, Gong M, Qin Y, Song H, Shi X, Wu Y, et al. Validation of glomerular filtration rate-estimating equations in Chinese children. PLoS One 2017;12:e0180565.
5. Kumar V, Yadav AK, Yasuda Y, Horio M, Kumar V, Sahni N, et al. Existing creatinine-based equations overestimate glomerular filtration rate in Indians. BMC Nephrol 2018;19:1-7.
6. Tang Z, Tao J, Sun L, Han Z, Chen H, Huang Z, et al. Prospective comparison of equations based on creatinine and cystatin C for the glomerular filtration rate estimation in Chinese renal transplant recipients. Transplant Proc 2018:85-91.
7. Yang M, Xu G, Ling L, Niu J, Lu T, Du X, et al. Performance of the creatinine and cystatin C-based equations for estimation of GFR in Chinese patients with chronic kidney disease. *Clin. Exp. Nephrol* 2017;21:236-246.
8. Chen M, Xia J, Pei G, Zhang Y, Wu S, Qin Y, et al. A more accurate method acquirement by a comparison of the prediction equations for estimating glomerular filtration rate in Chinese patients with obstructive nephropathy. BMC Nephrol 2016;17:1-10.
9. Changjie G, Xusheng Z, Feng H, Shuguang Q, Jianwen L, Junzhou F. Evaluation of glomerular filtration rate by different equations in Chinese elderly with chronic kidney disease *Int Urol Nephrol* 2017;49:133-141.
10. Guan C, Liang M, Liu R, Qin S, He F, Li J, et al. Assessment of creatinine and cystatin C-based eGFR equations in Chinese older adults with chronic kidney disease. *Int Urol Nephrol* 2018;50:2229-2238.
11. Huang Q, Sun X, Chen Y, Zhang M, Tang L, Liu S, et al. A study of the applicability of GFR evaluation equations for an elderly Chinese population. *J Nutr Health Aging* 2015;19:693-701.
12. Hu C, Li D, Yin W, Zuo X. Evaluation of cystatin C-derived glomerular filtration rate equations in Chinese population. Scand J Clin Lab Invest 2019;79:629-634.
13. Yang M, Zou Y, Lu T, Nan Y, Niu J, Du X, Gu Y, et al. Revised equations to estimate glomerular filtration rate from serum creatinine and cystatin C in China. *Kidney Blood Press Res* 2019;44:553-564.
14. Xie D, Shi H, Xie J, Ding Y, Zhang W, Ni L, et al. A validation study on eGFR equations in Chinese patients with diabetic or non-diabetic CKD. *Front Endocrinol* 2019;10:581.
15. Adachi M, Tanaka A, Aiso M, Takamori Y, Takikawa H. Benefit of cystatin C in evaluation of renal function and prediction of survival in patients with cirrhosis. Hepatol Res 2015;45:1299-1306.
16. Feng JF, Qiu L, Zhang L, Li XM, Yang YW, Zeng P, et al. Multicenter study of creatinine-and/or cystatin C-based equations for estimation of glomerular filtration rates in Chinese patients with chronic kidney disease. PLoS One 2013; 8:e57240.
17. Yue L, Pan B, Shi X, Du X. Comparison between the beta-2 microglobulin-based equation and the CKD-EPI equation for estimating GFR in CKD patients in China: ES-CKD study. Kidney Dis 2020;6:204-214.
18. Pei X, Yang W, Wang S, Zhu B, Wu J, Zhu J, et al. Using mathematical algorithms to modify glomerular filtration rate estimation equations. PLoS One 2013;8:e57852.

19. Feng JF, Qiu L, Zhang L, Li XM, Yang YW, Zeng P, et al. Multicenter study of creatinine-and/or cystatin C-based equations for estimation of glomerular filtration rates in Chinese patients with chronic kidney disease. PLoS One 2013; 8:e57240.
20. Horio M, Imai E, Yasuda Y, Watanabe T, Matsuo S, Collaborators Developing the Japanese Equation for Estimated GFR. GFR estimation using standardized serum cystatin C in Japan. *Am J Kidney Dis* 2013;61:197-203.
21. Teo BW, Xu H, Wang D, Li J, Sinha AK, Shuter B, et al. Estimating glomerular filtration rates by use of both cystatin C and standardized serum creatinine avoids ethnicity coefficients in Asian patients with chronic kidney disease. Clin Chem 2012;58:450-457.
