# Supplementary material for: Optimal Glomerular Filtration Rate Equations for Various Age Groups, Disease Conditions and Ethnicities in Asia: A Systematic Review
Source: J Clin Med. 2023 Feb 24;12(5):1822. doi: 10.3390/jcm12051822 (PMC10002889; doi:10.3390/jcm12051822)
Supplement: Supplementary file 1 [file jcm-12-01822-s001.zip › Supplementary Data Table S2.pdf]

Supplementary Data Table S2

Table S2 List of extracted equations with their abbreviations, expressions and references

No.	Name	Abbreviation	Equation	Reference
1.	Cockcroft-Gault	CG	(140-age) × Wt/72 × Scr × (0.85 if female)	[1]
2.	MDRD	MDRD	186 × $SCr^{-1.154}$× $age^{-0.203}$× (0.74 if female) × (1.21 if African)	[2]
3.	Re-expressed abbreviated MDRD or IDMS-MDRD	re-aMDRD or IDMS-MDRD	175 × $(Scr)^{-1.154}$ × $(Age)^{-0.203}$ × 0.742 (if female) × 1.212(if black)	[3]
4.	7MDRD	7MDRD	170 × $(Scr)^{-0.999}$ × (Age)$^{-0.176}$ × 0.762 (if female) × 1.180 (if black) × (BUN)$^{-0.170}$ × $(Alb)^{0.318}$	[4]
5.	Chinese-modified MDRD	c-MDRD	1.233 × 186 × $SCr^{-1.154}$× $age^{-0.203}$× (0.742 if female)	[5]
6.	American-modified MDRD	American-MDRD	186 × $SCr^{-1.154}$× $age^{-0.203}$× (0.742 if female)	[2]
7.	2009 CKD-EPI	CKD-EPI-Cr	141 × min (Scr(mg/dL)/k,1)a × max $(Scr(mg/dL)/k, 1)^{-1.209}$× 0.993^{age}× (1.018, if female) × (1.159, African) (female: k = 0.7, a = 0.329; male: k = 0.9, a = 0.411)	[6]
8.	2012 Cystatin-C-based CKD-EPI	CKD-EPI-CysC	133 ×min $(CysC/0.8, 1)^{-0.499}$×max $(CysC/0.8, 1)^{-1.328}$ × $0.996^{age\ (years)}$ (×0.932 if female)	[7]
9.	2012 Creatinine and Cystatin-C based CKD-EPI	CKD-EPI-Cr-CysC	135 × min $(Pcr/\kappa, 1)^{\alpha}$×max $(Pcr/\kappa, 1)^{-0.601}$× $min\ (CysC/0.8, 1)^{-0.375}$ ×max $(CysC/0.8, 1)^{-0.711}$× $0.995^{age\ (years)}$ × (0.969 if female) (κ is 0.7 for females and 0.9 for males; α is –0.248 for females and –0.207 for males.) × 1.08 (if African American)	[7]
10.	New Asian-modified CKD-EPI	EPI-Asian	male: eGFR = (2009 CKD-EPI) × 1.057; female: eGFR = (2009 CKD-EPI) × 1.049	[8]
11.	Pakistani CKD-EPI Equation	CKD-EPI-PK	0.686 × $(2009\ CKD - EPI)^{1.059}$	[9]
12.	Berlin Initiative Study equation 1	BIS-Cr or BIS-1	3726 × $SCr^{0.87}$ × $age^{-0.95}$ (×0.82, if female)	[10]
13.	Berlin Initiative	BIS-Cr-CysC or BIS-2	767 × $SCysC^{-0.61}$ × $SCr^{-0.40}$ × $age^{-0.157}$ (× 0.87, if female)	[10]

	Study equation 2			
14.	Japanese-modified MDRD	JSN-CKDI	$171 \times SCr^{-1.004} \times age^{-0.287} \times$ (0.782 if female)	[11]
15.	Japanese modified creatinine based CKD-EPI	Eq-Cr	0.813 × (2009 CKD-EPI)	[12]
16.	Japanese equation based on standardized SCysC	Eq-CysC	$96 \times CysC^{-1.324} \times 0.996^{age} \times$ 0.894 (if female)	[13]
17.	Japanese equation based on standardized SCr in combination with SCysC	Eq-Cr-CysC	$92 \times CysC^{-0.575} \times SCr^{-0.670} \times 0.995^{age} \times 0.784$ (if female)	[13]
18.	Japanese equation based on standardized SCysC with a nonrenal factor reflecting hypothesized extrarenal elimination	Eq-CysC-Nonrenal	{$104 \times CysC^{-1.019} \times 0.996^{age} \times$ 0.929 (if female)} – 8	[13]
19.	Larrson	Larrson	$77.24 \times CysC^{-1.2623}$(Dade Behring Cys C calibration)	[14]
20.	Rule	Rule	$76.6 \times CysC^{-1.16}$	[15]
21.	2003 Hoek Equation	2003 Hoek	80.35/Cys C - 4.32	[16]
22.	Filler	Filler	$91.62 \times CysC^{-1.123}$ Log (GFR) =1.962 + [1.123 × log (1/Cys C)])	[17]
23.	Grubb	Grubb	$99.19 \times CysC^{-1.713} \times$ (0.823 if female)	[18]
24.	Grubb Equation 2	Grubb 2	$84.64 \times CysC^{-1.686} \times$ (0.948, if female) × 1.384 (if a child <14 years)	[19]
25.	Creatinine-based Japanese Society of	eGFR-Cr	$194 \times SCr^{-1.904} \times age\ (years)^{-0.287}$(×0.739 if female)	[20]

	Nephrology equation			
26.	Cystatin-C-based Japanese Society of Nephrology equation	eGFR-CysC	$(104 \times CysC^{-1.019} \times 0.996^{age\ (years)}) - 8$ (male)<br>$(104 \times CysC^{-1.019} \times 0.996^{age\ (years)} \times 0.929) - 8$ (female)	[20]
27.	Revised Lund-Malmö Study equation	R-LM	$e^{X-0.0158\times Age+0.438\times \ln(Age)}$<br>Female pCr＜150μmol/L：X=2.50+0.0121×(150-pCr)<br>Female pCr≥150μmol/L：X=2.50-0.926×ln(pCr/150)<br>Male pCr＜180μmol/L：X=2.56+0.00968×(180-pCr)<br>Male pCr≥180μmol/L：X=2.56-0.926×ln(pCr/180)	[21]
28.	CAPA Equation	CAPA	$130 \times CysC^{-1.069} \times age^{-0.117} - 7$	[22]
29.	Creatinine-based Full Age Spectrum Equation	FAS-Cr	$107.3/(SCr/Q_{SCr}) \times [\ 0.988^{(Age-40)}$ when age＞40 years]<br>Q-values are the mean or median SCr value for age-/sex-specific healthy populations.	[23]
30.	Cystatin-C-based Full Age Spectrum Equation	FAS-CysC	$107.3/(CysC/Q_{CysC}) \times [\ 0.988^{(Age-40)}$ when age＞40 years]	[24]
31.	Combined Cystatin-C and Creatinine-based Full Age Spectrum Equation	FAS-Cr-CysC	$107.3/[\alpha \times (SCr/Q_{SCr}) + (1-\alpha) \times (CysC/Q_{CysC})\ ] \times [\ 0.988^{(Age-40)}$ when age＞40 years]	[24]
32.	MacIsaac Equation	MacIsaac	86.7 / CysC -4.2	[25]
33.	Pei (Modified MacIsaac) Equation	Pei (Modified MacIsaac)	77.30/CysC +2.32	[26]
34.	Feng Cystatin-C Equation	Feng-CysC	$78.64 \times CysC^{-0.964}$	[27]
35.	Feng Creatinine and Cystatin-C Equation	Feng-Cr-CysC	Female<br>$173.9 \times scr^{-0.184} \times cys^{-0.725} \times age^{-0.193} \times 0.89$<br>Male<br>$173.9 \times scr^{-0.184} \times cys^{-0.725} \times age^{-0.193}$	[27]
36.	1976 Schwartz Equation	Schwartz1976	$55 \times \frac{H}{SCrJ} \times 1.273^{boy>13yrs}$<br>H=Height in meters<br>SCrJ means creatinine measured by Jaffe method	[28]

37.	2009 Schwartz Equation	Schwartz2009	$41.3\times\frac{H}{SCrE}$<br>H=Height in meters<br>ScrE means creatinine measured by IDMS-traceable enzymatic method	[29]
38.	Chronic Kidney Disease in Children Study Equation	CKiD	$39.1\times\frac{H}{SCrE}^{0.516}\times\frac{1.8}{CysC}^{0.294}\times\frac{30}{BUN}^{0.169}\times[1.099^{boy}]\times\frac{H}{1.4}^{0.188}$<br>H=Height in meters<br>BUN= blood urea Nitrogen<br>ScrE means creatinine measured by IDMS-traceable enzymatic method	[29]
39.	Modified CKD-EPI Equation by Pei	Modified CKD-EPI by Pei	Female ≤ 62μmol/liter<br>$144\times\frac{SCr}{0.7}^{0.156}\times 0.993^{age}$<br>Female ≥ 62μmol/liter<br>$144\times\frac{SCr}{0.7}^{-1.057}\times 0.993^{age}$<br>Male ≤ 80μmol/liter<br>$141\times\frac{SCr}{0.9}^{0.074}\times 0.993^{age}$<br>Male ≤ 80μmol/liter<br>$141\times\frac{SCr}{0.9}^{-1.057}\times 0.993^{age}$	[26]
40.	Modified MDRD Equation by Pei	Modified MDRD by Pei	$186\times SCr^{-0.830}\times Age^{-0.230}\times$ (0.742 if female)	[26]
41.	Chinese new creatinine-based MDRD Equation	Ma-Cr	Female<br>$175\times(scr)^{-1.234}\times age^{-0.179}\times 0.79$<br>Male<br>$175\times(scr)^{-1.234}\times age^{-0.179}$	[5]
42.	Chinese new cystatin C based Equation	Ma-CysC	$86\times cys^{-1.132}$	[30]
43.	Chinese new creatinine and cystatin-C based Equation	Ma-Cr-CysC	Female<br>$169\times scr^{-0.608}\times cys^{-0.63}\times age^{-0.157}\times 0.83$<br>Male<br>$169\times scr^{-0.608}\times cys^{-0.63}\times age^{-0.157}$	[30]
44.	Chinese new creatinine and cystatin-C based Equation	Ma-Cr-CysC Equation 1	$176\times SCr^{-0.607}\times SCysC^{-0.638}\times age^{-0.171}$<br>(×0.85,if female)	[30]
45.	Beta-2 microglobulin Equation	B2M	$133\times B2M^{-0.852}$	[31]

46.	Yang Du Creatinine-based Equation	C-CKD-EPI-Cr	Female ≤ 0.7<br>$125 \times \frac{SCr^{-0.329}}{0.7} \times 0.993^{Age}$<br>Female ≥ 0.7<br>$125 \times \frac{SCr^{-1.209}}{0.7} \times 0.993^{Age}$<br>Male ≤ 0.9<br>$123 \times \frac{SCr^{-0.411}}{0.9} \times 0.993^{Age}$<br>Male ≥ 0.9<br>$123 \times \frac{SCr^{-1.209}}{0.9} \times 0.993^{Age}$	[32]
47.	Yang Du Cystatin-C based Equation	C-CKD-EPI-CysC	≤ 0.8<br>$117 \times \frac{SCysC)^{-0.499}}{0.8} \times 0.996^{age}$<br>≥ 0.8<br>$117 \times \frac{SCysC)^{-1.328}}{0.8} \times 0.996^{age}$	[32]
48.	Yang Du Creatinine and Cystatin-C based Equation	C-CKD-EPI-Cr-CysC	Female Scr ≤ 0.7 SCysC ≤ 0.8<br>$116 \times \frac{Scr^{-0.248}}{0.7} \times \frac{SCysC)^{-0.375}}{0.8} \times 0.996^{age}$<br>Female Scr ≤ 0.7 SCysC > 0.8<br>$116 \times \frac{Scr^{-0.248}}{0.7} \times \frac{SCysC)^{-0.711}}{0.8} \times 0.996^{age}$<br>Female Scr > 0.7 SCysC ≤ 0.8<br>$116 \times \frac{Scr^{-0.601}}{0.7} \times \frac{SCysC)^{-0.375}}{0.8} \times 0.996^{age}$<br>Female Scr > 0.7 SCysC > 0.8<br>$116 \times \frac{Scr^{-0.601}}{0.7} \times \frac{SCysC)^{-0.711}}{0.8} \times 0.996^{age}$<br>Male Scr ≤ 0.9 SCysC ≤ 0.8<br>$120 \times \frac{Scr^{-0.207}}{0.9} \times \frac{SCysC)^{-0.375}}{0.8} \times 0.996^{age}$<br>Male Scr ≤ 0.9 SCysC > 0.8<br>$120 \times \frac{Scr^{-0.207}}{0.9} \times \frac{SCysC)^{-0.711}}{0.8} \times 0.996^{age}$<br>Male Scr > 0.9 SCysC ≤ 0.8<br>$120 \times \frac{Scr^{-0.601}}{0.9} \times \frac{SCysC)^{-0.375}}{0.8} \times 0.996^{age}$<br>Male Scr > 0.9 SCysC > 0.8<br>$120 \times \frac{Scr^{-0.601}}{0.9} \times \frac{SCysC)^{-0.711}}{0.8} \times 0.996^{age}$	[32]
49.	Xiangya Equation	Xiangya	Male<br>$2{,}374.78 \times SCr^{-0.54753} \times Age^{-0.25011}$<br>Female<br>$2{,}374.78 \times SCr^{-0.54753} \times Age^{-0.25011} \times 0.8526126$	[33]
50.	CKD-EPI Equations Re-expressed for Standardized SCysC	Standardized SCysC CKD-EPI Equation 1	$76.7 \times (-0.105 + 1.13 \times \text{standardized SCysC})^{-1.19}$	[34, 35]

51.	CKD-EPI Equations Re-expressed for Standardized SCysC	Standardized SCysC CKD-EPI Equation 2	$127.7 \times (-0.105 + 1.13 \times \text{standardized SCysC})^{-1.17} \times \text{age}^{-0.13} \times (0.91 \text{ if female}) \times (1.06 \text{ if black})$	[34, 35]
52.	CKD-EPI Equations Re-expressed for Standardized SCr and SCysC	Standardized SCr and SCysC CKD-EPI	$177.6 \times \text{standardized SCr}^{-0.65} \times (-0.105 + 1.13 \times \text{standardized SCysC})^{-0.57} \times \text{age}^{-0.20} \times (0.82 \text{ if female}) \times (1.11 \text{ if black})$	[34, 35]
53.	Modified Cystatin-C based CKD-EPI Equation for elderly	Modified CKD-EPI-CysC for elderly	Female SCysC≤ 0.8<br>$133 \times (sCysC/0.8)^{-0.499} \times 0.996^{age} \times 0.932$<br>Female SCysC ≥ 0.8<br>$134 \times (sCysC/0.8)^{-0.899} \times 0.996^{age} \times 0.874$<br>Male SCysC ≤ 0.8<br>$133 \times (sCysC/0.8)^{-0.499} \times 0.996^{age}$<br>Male SCysC ≥ 0.8<br>$120 \times (sCysC/0.8)^{-0.825} \times 0.996^{age}$	[36]
54.	Modified Creatinine and Cystatin-C based CKD-EPI Equation for elderly	Modified CKD-EPI-Cr and CysC for elderly	Females:SCr ≤ 0.7<br>If SCysC ≤ 0.8<br>$130 \times \frac{SCr}{0.7}^{-0.248} \times \frac{SCysC}{0.8}^{-0.375} \times 0.995^{age} (\times 1.08 \text{ if Black})$<br>If SCysC ≥ 0.8<br>$130 \times \frac{SCr}{0.7}^{-0.248} \times \frac{SCysC}{0.8}^{-0.711} \times 0.995^{age} (\times 1.08 \text{ if Black})$<br>SCr ≥ 0.7<br>If SCysC ≤ 0.8<br>$130 \times \frac{SCr}{0.7}^{-0.601} \times \frac{SCysC}{0.8}^{-0.375} \times 0.995^{age} (\times 1.08 \text{ if Black})$<br>If SCysC ≥ 0.8<br>$130 \times \frac{SCr}{0.7}^{-0.601} \times \frac{SCysC}{0.8}^{-0.711} \times 0.995^{age} (\times 1.08 \text{ if Black})$<br>Males: SCr ≤ 0.9<br>If SCysC ≤ 0.8<br>$135 \times \frac{SCr}{0.9}^{-0.207} \times \frac{SCysC}{0.8}^{-0.375} \times 0.995^{age} (\times 1.08 \text{ if Black})$<br>If SCysC ≥ 0.8<br>$135 \times \frac{SCr}{0.9}^{-0.207} \times \frac{SCysC}{0.8}^{-0.711} \times 0.995^{age} (\times 1.08 \text{ if Black})$<br>SCr ≥0.9<br>If SCysC ≤ 0.8<br>$135 \times \frac{SCr}{0.9}^{-0.601} \times \frac{SCysC}{0.8}^{-0.375} \times 0.995^{age} (\times 1.08 \text{ if Black})$	[36]

			If SCysC ≥ 0.8<br>$135 \times \frac{SCr}{0.9}^{-0.601} \times \frac{SCysC}{0.8}^{-0.711} \times 0.995^{age}$ (×1.08 if Black)	

References:

1. Cockcroft DW and Gault H. Prediction of creatinine clearance from serum creatinine. Nephron 1976;16:31-41.
2. Levey A, Greene T, Kusek J, Beck G, Group MS. A simplified equation to predict glomerular filtration rate from serum creatinine. J Am Soc Nephrol 2000;11(S2):155.
3. Levey AS, Coresh J, Greene T, Marsh J, Stevens LA, Kusek JW, et al. Expressing the Modification of Diet in Renal Disease Study equation for estimating glomerular filtration rate with standardized serum creatinine values. Clin Chem 2007;53:766-772.
4. Levey AS, Bosch JP, Lewis JB, Greene T, Rogers N, Roth D. A more accurate method to estimate glomerular filtration rate from serum creatinine: a new prediction equation. Ann Intern Med 1999;130:461-470.
5. Ma YC, Zuo L, Chen JH, Luo Q, Yu XQ, Li Y, et al. Modified glomerular filtration rate estimating equation for Chinese patients with chronic kidney disease. J Am Soc Nephrol 2006;17:2937-2944.
6. Levey AS, Stevens LA, Schmid CH, Zhang Y, Castro III AF, Feldman HI, et al. A new equation to estimate glomerular filtration rate. Ann Intern Med 2009;150:604-612.
7. Inker LA, Schmid CH, Tighiouart H, Eckfeldt JH, Feldman HI, Greene T, et al. Estimating glomerular filtration rate from serum creatinine and cystatin C. N Engl J Med 2012;367:20-29.
8. Stevens LA, Claybon MA, Schmid CH, Chen J, Horio M, Imai E, et al. Evaluation of the Chronic Kidney Disease Epidemiology Collaboration equation for estimating the glomerular filtration rate in multiple ethnicities. Kidney Int 2011;79:555-562.
9. Jessani S, Levey AS, Bux R, Inker LA, Islam M, Chaturvedi N, et al. Estimation of GFR in South Asians: a study from the general population in Pakistan. Am J Kidney Dis 2014; 63:49-58
10. Schaeffner ES, Ebert N, Delanaye P, Frei U, Gaedeke J, Jakob O, et al. Two novel equations to estimate kidney function in persons aged 70 years or older. Ann Intern Med 2012;157:471-481.
11. Imai E, Horio M, Nitta K, Yamagata K, Iseki K, Tsukamoto Y, et al. Modification of the modification of diet in renal disease (MDRD) study equation for Japan. Am J Kidney Dis 2007;50:927-937.
12. Horio M, Imai E, Yasuda Y, Watanabe T, Matsuo S. Modification of the CKD epidemiology collaboration (CKD-EPI) equation for Japanese: accuracy and use for population estimates. Am J Kidney Dis 2010;56:32-38.
13. Horio M, Imai E, Yasuda Y, Watanabe T, Matsuo S, Collaborators Developing the Japanese Equation for Estimated GFR. GFR estimation using standardized serum cystatin C in Japan. Am J Kidney Dis 2013;61:197-203.
14. Larsson A, Malm J, Grubb A, Hansson L-O. Calculation of glomerular filtration rate expressed in mL/min from plasma cystatin C values in mg/L. Scand J Clin Lab Invest 2004;64(1):25-30.
15. Rule A, Bergstralh E, Slezak J, Bergert J, Larson T. Glomerular filtration rate estimated by cystatin C among different clinical presentations. Kidney Int 2006;69:399-405.
16. Hoek FJ, Kemperman FA, Krediet RT. A comparison between cystatin C, plasma creatinine and the Cockcroft and Gault formula for the estimation of glomerular filtration rate. Nephrol Dial Transplant 2003;18:2024-2031.
17. Filler G and Lepage N. Should the Schwartz formula for estimation of GFR be replaced by cystatin C formula? Pediatr Nephrol 2003;18:981-985.
18. Grubb A, Björk J, Lindström V, Sterner G, Bondesson P, Nyman U. A cystatin C-based formula without anthropometric variables estimates glomerular filtration rate better than creatinine clearance using the Cockcroft-Gault formula. Scand J Clin Lab Invest 2005;65:153-162.
19. Grubb A, Nyman U, Björk J, Lindström V, Rippe B, Sterner G, et al. Simple cystatin C–based prediction equations for glomerular filtration rate compared with the modification of diet in renal disease prediction equation for adults and the Schwartz and the Counahan–Barratt prediction equations for children. Clin Chem 2005;51:1420-1431.
20. Nihon Jinzo Gakkai shi. Clinical practice guidebook for diagnosis and treatment of CKD. Japanese Society of Nephrology 2007; 49:757-861

21. Nyman U, Grubb A, Larsson A, Hansson LO, Flodin M, Nordin G, et al. The revised Lund-Malmö GFR estimating equation outperforms MDRD and CKD-EPI across GFR, age and BMI intervals in a large Swedish population. Clin Chem Lab Med 2014;52:815-824.
22. Grubb A, Horio M, Hansson LO, Björk J, Nyman U, Flodin M, et al. Generation of a new cystatin C–based estimating equation for glomerular filtration rate by use of 7 assays standardized to the international calibrator. Clin Chem 2014;60:974-986.
23. Pottel H, Hoste L, Dubourg L, Ebert N, Schaeffner E, Eriksen BO, et al. An estimated glomerular filtration rate equation for the full age spectrum. Nephrol Dial Transplant 2016;31:798-806.
24. Pottel H, Delanaye P, Schaeffner E, Dubourg L, Eriksen BO, Melsom T, et al. Estimating glomerular filtration rate for the full age spectrum from serum creatinine and cystatin C. Nephrol Dial Transplant 2017;32:497-507.
25. Macisaac RJ, Tsalamandris C, Thomas MC, Premaratne E, Panagiotopoulos S, Smith TJ, et al. Estimating glomerular filtration rate in diabetes: a comparison of cystatin-C-and creatinine-based methods. Diabetologia. 2006;49:1686-1689.
26. Pei X, Yang W, Wang S, Zhu B, Wu J, Zhu J, et al. Using mathematical algorithms to modify glomerular filtration rate estimation equations. PLoS One 2013;8:e57852.
27. Feng JF, Qiu L, Zhang L, Li XM, Yang YW, Zeng P, et al. Multicenter study of creatinine-and/or cystatin C-based equations for estimation of glomerular filtration rates in Chinese patients with chronic kidney disease. PLoS One 2013;8:e57240.
28. Schwartz G, Haycock G, Edelmann Jr C, Spitzer A. A simple estimate of glomerular filtration rate in children derived from body length and plasma creatinine. Pediatrics 1976;58:259-263.
29. Schwartz GJ, Munoz A, Schneider MF, Mak RH, Kaskel F, Warady BA, et al. New equations to estimate GFR in children with CKD. J Am Soc Nephrol 2009;20:629-637.
30. Ma YC, Zuo L, Chen JH, Luo Q, Yu XQ, Li Y, et al. Improved GFR estimation by combined creatinine and cystatin C measurements. Kidney Int 2007;72:1535-1542.
31. Inker LA, Tighiouart H, Coresh J, Foster MC, Anderson AH, Beck GJ, et al. GFR estimation using β-trace protein and β2-microglobulin in CKD. Am J Kidney Dis 2016;67:40-48.
32. Yang M, Zou Y, Lu T, Nan Y, Niu J, Du X, et al. Revised equations to estimate glomerular filtration rate from serum creatinine and cystatin C in China. Kidney Blood Press Res 2019;44:553-564.
33. Li DY, Yin WJ, Yi YH, Zhang BK, Zhao J, Zhu CN, et al. Development and validation of a more accurate estimating equation for glomerular filtration rate in a Chinese population. Kidney Int 2019;95:636-646.
34. Inker LA, Eckfeldt J, Levey AS, Leiendecker-Foster C, Rynders G, Manzi J, et al. Expressing the CKD-EPI (Chronic Kidney Disease Epidemiology Collaboration) cystatin C equations for estimating GFR with standardized serum cystatin C values. Am J Kidney Dis 2011;58:682-684.
35. Stevens LA, Coresh J, Schmid CH, Feldman HI, Froissart M, Kusek J, et al. Estimating GFR using serum cystatin C alone and in combination with serum creatinine: a pooled analysis of 3,418 individuals with CKD. Am J Kidney Dis 2008;51:395-406.
36. Li F, Pei X, Ye X, Liu X, Song D, Zhang X, et al. Modification of the 2012 CKD-EPI equations for the elderly Chinese. Int Urol Nephrol 2017;49:467-473.
